# Lightweight Gas Sensor Based on MEMS Pre-Concentration and Infrared Absorption Spectroscopy Inside a Hollow Fiber

**DOI:** 10.3390/s23052809

**Published:** 2023-03-03

**Authors:** Roberto Viola, Nicola Liberatore, Sandro Mengali, Ivan Elmi, Fabrizio Tamarri, Stefano Zampolli

**Affiliations:** 1 Centro Ricerche Elettro Ottiche, 67100 L’Aquila, Italy; 2 Institute for Microelectronics and Microsystems, Italian National Research Council CNR-IMM, 40129 Bologna, Italy

**Keywords:** gas analyzer, MEMS, absorption spectroscopy, IR hollow fiber, safety and security

## Abstract

This paper reports on a compact and lightweight sensor for analysis of gases/vapors by means of a MEMS-based pre-concentrator coupled to a miniaturized infrared absorption spectroscopy (IRAS) module. The pre-concentrator was utilized to sample and trap vapors in a MEMS cartridge filled with sorbent material and to release them once concentrated by fast thermal desorption. It was also equipped with a photoionization detector for in-line detection and monitoring of the sampled concentration. The vapors released by the MEMS pre-concentrator are injected into a hollow fiber, which acts as the analysis cell of the IRAS module. The miniaturized internal volume of the hollow fiber of about 20 microliters keeps the vapors concentrated for analysis, thus allowing measurement of their infrared absorption spectrum with a signal to noise ratio high enough to identify the molecule, despite the short optical path, starting from sampled concentration in air down to parts per million. Results obtained for ammonia, sulfur hexafluoride, ethanol and isopropanol are reported to illustrate the sensor detection and identification capability. A limit of identification (LoI) of about 10 parts per million was validated in the lab for ammonia. The lightweight and low power consumption design of the sensor allowed operation onboard unmanned aerial vehicles (UAVs). The first prototype was developed within the EU Horizon 2020 project ROCSAFE for the remote assessment and forensic examination of a scene in the aftermath of industrial or terroristic accidents.

## 1. Introduction

In the aftermath of industrial accidents or terroristic attacks involving chemical–biological–radiological–nuclear–explosive (CBRNE) materials, the availability of point sensors to be deployed on the field onboard unmanned aerial vehicles and robotic ground vehicles (RGVs) in order to reveal and rapidly identify toxic compounds that are present in the area could avoid exposing operative personnel to the threat [1,2,3]. Even though several portable products and standoff sensors are available on the market, in particular for detection of chemical threats [4,5], currently there are no specific sensors available to be easily deployed at a fair cost and capable of running autonomously to survey the presence of such chemical compounds inside a suspected area. Moreover, innovative methods for gas sensing based on plasmonic metasurfaces are known from the literature [6,7,8,9], which are highly sensitive and operable in real time. However, they are not yet consolidated, nor at sufficient technology readiness level (TRL), to be adopted for security and forensic applications in particular. Regarding point sensors, they should be operated in local but distributed networks, and data gathered by such networks should be evaluated by expert operators. Such monitoring activities are necessary both to assess the actual conditions and prevent hazards for the personnel that will access the site, and to collect information useful for forensic activities. Common characteristics of this scenario should be taken into account: (a) the range of chemical targets is wide [10]; (b) events of interest may have a rapid time evolution (in the order of minutes, or even seconds), because the sensor might cross through a localized gas cloud onboard a UAV at non-negligible speed, or gas clouds might spread towards different directions depending on variable weather conditions [11]; (c) concentration of target molecules may be low, and usually a fraction of saturation concentration [12]; (d) even for gases and substances with high vapor pressure, the concentration can decrease rapidly when the gas cloud diffuses over the accident site; (e) interferents could be present, that may mask the signals of the target, thus decreasing the probability of detection (PoD), or they may mimic the signals of the target, thus increasing the probability of false alarm (PFA) [13,14]. In order to overcome all these issues, the sensors should be capable of detecting several compounds and distinguishing them from each other, the response should be rapid, the detection limit should be appreciably lower than the saturation concentration of targeted compounds, and the algorithms for identification should be able to reject signals of interfering compounds. We present a lightweight sensor (LIRAS) based on infrared absorption spectroscopy inside a hollow fiber [15,16,17], which was developed within the EU Horizon 2020 Programme for the Project ROCSAFE [18] to be operated onboard UAVs or small RGVs to identify traces of chemical warfare agents (CWAs) and toxic industrial compounds (TICs) present in the environment surrounding an accident area. Moreover, a possible use of this sensor is in the analysis of the vapors over the surface of a puddle, or in the headspace of an open vessel, to discriminate, for example, water from a liquid of forensic interest.

## 2. Sensor Description

**Figure 1:**
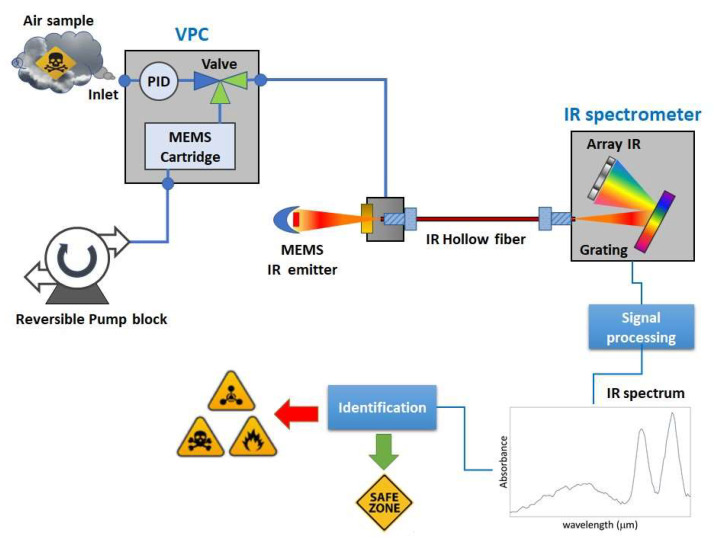
Concept design of the LIRAS sensor.

The concept of the LIRAS sensor is depicted in Figure 1.

It is a compact sensor based on a lightweight IR dispersive analyzer connected with a vapor sampling and pre-concentration module (VPC). The VPC couples a MEMS-based pre-concentration cartridge [19] packed with a suitable absorbent material to a pump block managing the sample acquisition and injection, and to a commercial photoionization detector (PID) used to track the amount of pre-concentrated sample. The IR analyzer consists of a MEMS thermal source, an IR hollow fiber, and a miniaturized spectrometer, plus electronic boards and mechanical/fluidic/electrical interfaces. The hollow fiber acts as an optical cell, in which the vapors, previously concentrated in the VPC module, are released for the analysis. The IR radiation from the MEMS thermal source is injected into one end of the hollow fiber. At the other end of the fiber, the transmitted radiation is dispersed onto an array of detectors, which allows it to determine the IR absorption spectrum of the vapor. The sensor can work in two operation modes: concentration monitoring, or analysis. In the concentration monitoring mode, useful to estimate the total amount of sampled vapors, the PID makes a concentration measurement every second, while the VPC is sampling and the IR analyzer is idle. In the analysis mode, sampling is stopped and the VPC thermally desorbs and injects pre-concentrated vapors into the IR analyzer, while the PID is idle. The specifications of the LIRAS sensor prototype developed in ROCSAFE are reported in Table 1.

### 2.1. Sampling and Pre-Concentration Module 

The sampling and pre-concentration module allows direct sampling of the air and is used to capture and concentrate vapors before the analysis to improve the sensitivity of the IRAS sensor. It is composed of: (a) pre-concentrator module implementing a PID for total volatile organic compound (VOC) mapping and a MEMS pre-concentration cartridge for capture and desorption of vapors; (b) sampling pump module implementing a reversible pump system, capable of providing a high sampling flow and a low injection flow. The photoionization detector is the MiniPID 2 from ION Science Ltd., Fowlmere, United Kingdom, which utilizes a 10.6 eV lamp. It is a low-power, small, ATEX-certified photoionization detector featuring a patented fence-electrode design to minimize background noise and interference by humidity. The specifications of the PID are reported in Table 2. 

**Figure 2:**
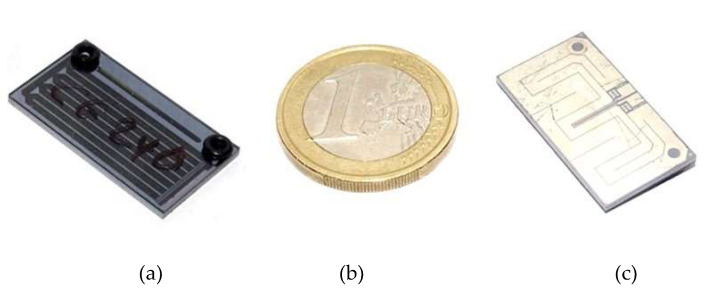
MEMS pre-concentration cartridge. (**a**) bottom view, (**b**) 1 euro coin for size comparison, (**c**) top view.

The MEMS pre-concentration cartridge [19] consists of a micro-channel etched in a silicon/Pyrex stack and packed with a suitable absorbent mesh. On the backside of the silicon wafer a platinum metallization implements a temperature sensor and a heater resistor for direct heating of the pre-concentrator chip by means of a custom designed electronic circuit. The cartridges are 25 × 14 × 1.5 mm^3^ and weigh about 1 g after packing with the active material. Figure 2 shows photographs of the MEMS pre-concentration cartridge developed and optimized for the LIRAS pre-concentrator, with a EUR 1 coin for size comparison. 

**Figure 3:**
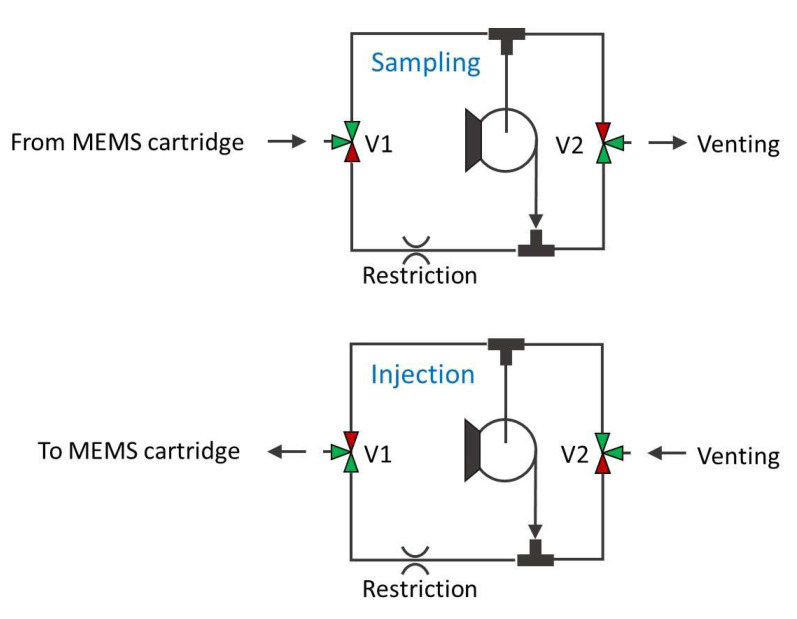
Schematics of the fluidic circuit of the sampling pump module.

The cartridge was filled with Carbograph 240 m^2^/g, which is suitable for capturing highly volatile species. The efficiency of a purge and trap thermal pre-concentration cycle depends on a number of variables and varies with different analytes, sorbent material type, and mass. In particular, the sorbent needs to be optimized for the target analytes, with highly volatile gases requiring stronger sorbents than low-volatile or high-boiling vapors. The main characteristic figure of a sorbent material is the breakthrough volume, indicating the volume of carrier gas necessary to purge a specific analyte through 1 g of sorbent at a specific temperature. Ideally, if the best sorbent is chosen for a specific analyte and kept at a suitable low temperature to avoid reaching the breakthrough volume, the theoretical pre-concentration coefficient is the ratio between the sampled volume and the volume of the carrier gas used for complete analyte desorption. In reality, the main limitation of the MEMS pre-concentrator used in this work is the small amount of sorbent, typically around 40 mg, resulting in a rather low breakthrough volume and thus lowering the effective pre-concentration coefficient. In a previous work, the pre-concentration effect for different target compounds was compared, using similar MEMS devices coupled to a GC/IMS for detection [19]. In any case, the use of the proposed LIRAS system is the detection and identification of specific target gases at low concentrations, and not their quantitative measurement, therefore the exact determination of pre-concentration coefficient and linearity for the different target gases is beyond the scope of this work. Figure 3 shows the fluidic schematic circuit implemented in the sampling pump module during the sampling phase and injection phase.

In the injection phase, the pump provides overpressure through a suitable restriction, which was obtained by a few centimeters of capillary tubing with 100 μm internal diameter, resulting in a low flow (a few mL/min) into the pre-concentrator module. The same circuit allows reversal of the flow direction in the pre-concentrator during the sampling phase, where both 3-way valves (V1 and V2 in Figure 3) are switched and the pump underpressure is provided without any restrictions to the pre-concentrator module for high flow sampling into the MEMS pre-concentrator cartridge (about 100 mL/min). The SMC pneumatics model S070C-SAG-32 was identified as most suitable 3-port solenoid valve, due to its small size, small dimensions, low weight, and low price. The specifications of this valve are reported in Table 3. Since the sensor is intended to give a prompt alarm if hazardous analytes are identified, the presence of the analyte in the air used as a carrier for injection and the release of the concentrated analyte in the environment after the analysis are not an issue.

Two specifically designed electronic boards based on a micro-controller, one for the MEMS temperature control (TC-PCB), one for PID signal acquisition (PID-PCB), and specific firmware capable of applying fixed set-point or temperature ramps on the MEMS pre-concentrator were developed. They share the I2C communication bus and protocols for easy integration with the rest of the LIRAS sensor electronics. The TC-PCB micro-controller board was used to control the temperature of the pre-concentrator and its metallic interconnect manifold, and to drive the valves and the pump, plus a cooling fan to cool down the MEMS cartridge before and during the sampling phase. The PID-PCB micro-controller board was used to acquire the signal of the mini-PID detector using a 16-bit A/D converter. The driving electronics allowed for switching the PID detector lamp on and off, to increase PID lamp lifetime and reduce power consumption. Furthermore, self-test capabilities and auto-calibration functionalities were also implemented.

### 2.2. IR Dispersive Analyzer

The IR dispersive analyzer utilizes infrared absorption spectroscopy, which is recognized as one of the best analysis techniques for identification of molecules in the vapor phase, for the analysis of the vapors captured by VPC. A MEMS thermal emitter, namely the EMIRS IR-55 from Axetris AG, Kaegiswil, Switzerland, was selected as the lightweight and low power consumption compact source to generate IR radiation over a wide spectral range. It combines high emissivity in the IR spectral range with low power consumption to achieve a working temperature of 450 °C. It was electrically modulated at 7 Hz to improve signal to noise ratio by synchronous detection. The source is also equipped with a small parabolic reflector, which confines the radiation within a small angle of emission to increase the amount of radiation injected into the optical fiber. The specifications of the source are given in Table 4.

**Figure 4:**
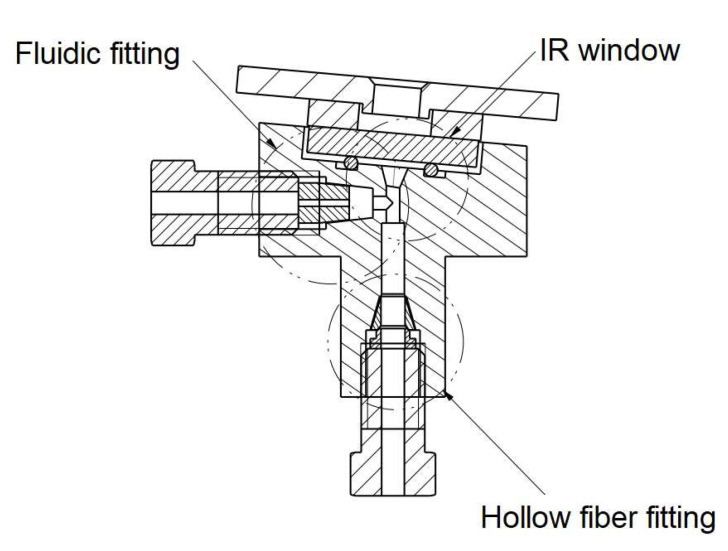
Section of the opto-fluidic interface cell.

A hollow optical fiber acted as the absorption cell of the sensor, since it allows both the radiation from the IR source and the vapors desorbed by the VPC to pass through. An opto-fluidic cell of miniaturized internal volume was purposely developed to interface the hollow fiber with the MEMS IR source through an infrared window, and with the fluidic outlet of the VPC by means of a VICI Valco 1/16” nut, as shown in Figure 4. 

The broad spectral emission of the thermal source is selectively absorbed inside the hollow fiber by the vapors, thus revealing their identity. We used the hollow optical fiber by OptoKnowledge, which is a silica tube with an external protective cladding and an internal reflective coating, and a ZnSe window for the opto-fluidic cell. The main specifications of the hollow optical fiber are reported in Table 5. 

In the custom designed micro-spectrometer, a diffraction grating was used to disperse the IR radiation coming from the hollow fiber onto a linear array of thermal detectors (LDA), in such a way that each element of the array collects the radiation at a different wavelength, thus allowing retrieval of the absorption spectra. The specifications of the small grating by Thorlabs implemented in the micro-spectrometer of our LIRAS prototype are reported in Table 6.

A linear detector array collects the IR radiation dispersed by the grating of the micro-spectrometer. The linear size and pitch of the detector array were selected according to the grating dispersion to acquire a wavelength range useful for identification of several targeted compounds. A linear array of pyroelectric detectors, namely the model Pyrosens by DIAS Infrared Systems, whose specifications are reported in Table 7, was finally implemented to acquire a spectral range of about 2.5 μm over 128 spectral channels with a spectral resolution of about 0.15 μm. 

A micro-controller board, model MSP-432P401R by Texas Instruments, which is based on a 32-bit micro-controller with ARM core, was used to manage the I2C communication to control the IR MEMS emitter and acquire the signals of the linear detector. 

**Figure 5:**
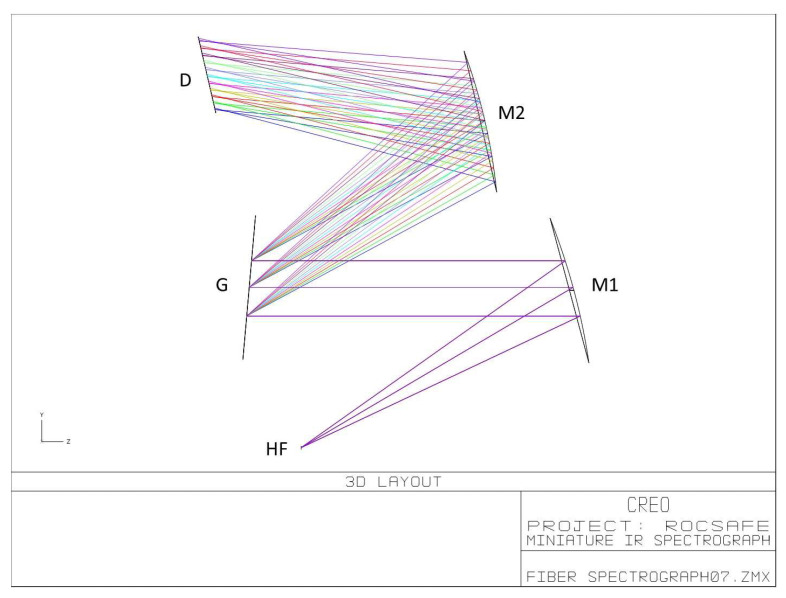
Optical scheme of the miniature IR spectrograph, where HF is the exit aperture of the hollow fiber; M1 is a 30 deg. off-axis parabolic mirror; M2 is a spherical mirror; G is the diffraction grating; D is the sensitive surface of the LDA.

Bearing in mind the requirement of implementation onboard UAVs, the Czerny–Turner configuration was adopted for pursuing the compactness of the IR spectrograph. Moreover, in order to reduce costs and time for development, the selection of optics was driven by market availability of commercial off-the-shelf (COTS) components. The specifications of selected COTS components were used in the simulation of the optical system using the Zemax optical design program. Figure 5 reports the 3D layout of the simulated optical system, where the exit aperture of the hollow fiber coincides with the entrance aperture of the spectrometer. 

**Figure 6:**
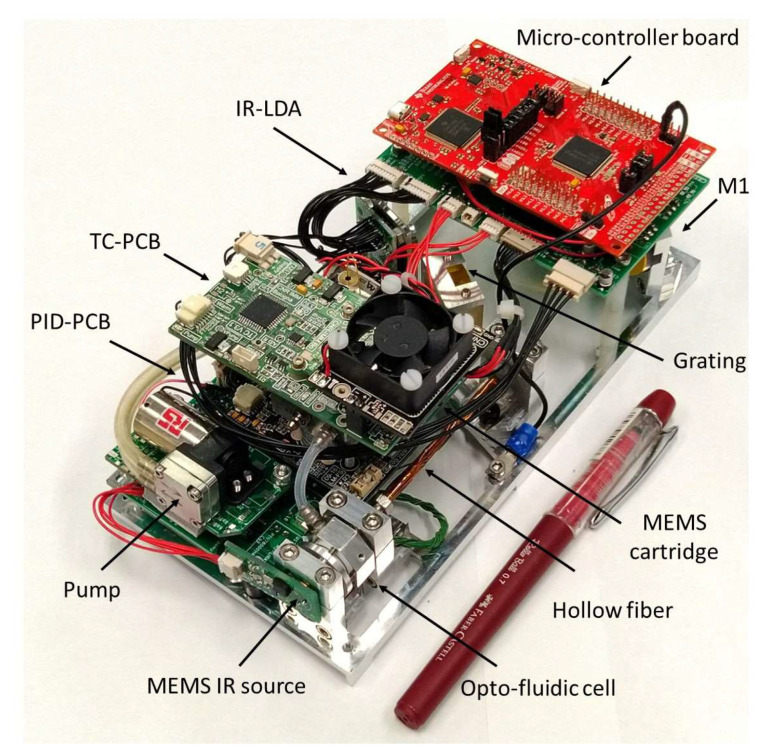
LIRAS sensor prototype.

The optical components were selected by trading off the system performance in terms of spectral range and resolution with the compactness in the simulations. In the actual LIRAS prototype, the IR radiometric signal emerging from the hollow fiber is spread over the LDA in the spectral range of wavelengths between 8.5 μm and 11 μm, where IR spectral fingerprints of several molecules are present, including toxic industrial TICs and CWAs [20]. Figure 6 shows the LIRAS sensor prototype developed in ROCSAFE, with a pen for size comparison, where the IR analyzer and the pre-concentrator module were assembled together in a compact device weighing about 0.5 kg.

The LIRAS sensor was also equipped with a Wi-Fi transmission unit, and a graphical user interface (GUI) running on a portable PC, tablet, or smartphone was developed. It can remotely launch a pre-defined procedure of sampling and analysis and show in real time detection and identification results if they occur, by using runtime algorithms of correlation to find the best match between the spectra measured (approximately every 2 s) and the spectra from a reference database. The remote control was successfully demonstrated outdoors at a short distance in the final demo of the project ROCSAFE. Of course, an alternative Wi-Fi solution could be implemented to run the sensor remotely in field conditions and at longer distances.

## 3. Results and Discussion

**Figure 7:**
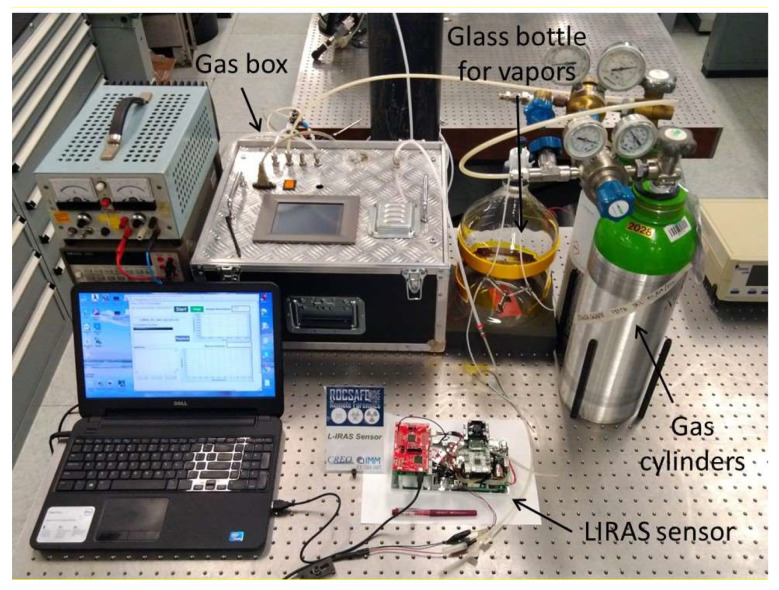
Laboratory setup for testing of the LIRAS sensor.

Figure 7 shows a picture of the test bench set up to carry out several tests with real compounds at different concentration levels. For testing with gases, different standard pressurized gas cylinders were utilized. The first one contained a reference mix of ammonia (NH_3_) at the concentration of 1000 ppm (parts per million) in nitrogen, the second one contained a reference mix of sulfur hexafluoride (SF_6_) at the concentration of 1000 ppm in nitrogen, and the third one was filled with pure nitrogen. They were connected to a gas box equipped with mass flow controllers and switching valves allowing the production of controlled mixes at diluted concentrations to investigate the response of the sensor and evaluate its limit of detection (LoD) for compounds of interest in security applications. Ammonia was at the top of the list of hazardous chemicals provided by the International Task Force-25 [10]. Sulfur hexafluoride is a safe simulant of volatile nerve agents [21]. For testing with safe vapors of liquid compounds at room temperature, glass bottles containing liquid VOC standards, in particular ethanol (CAS# 64-17-5) and isopropanol (CAS# 67-63-0) were procured from Carlo Erba Reagents, Cornaredo, Italy, and small amounts of liquid were evaporated into a 5.5-L glass bottle to produce controlled vapor concentration assuming total evaporation.

**Figure 8:**
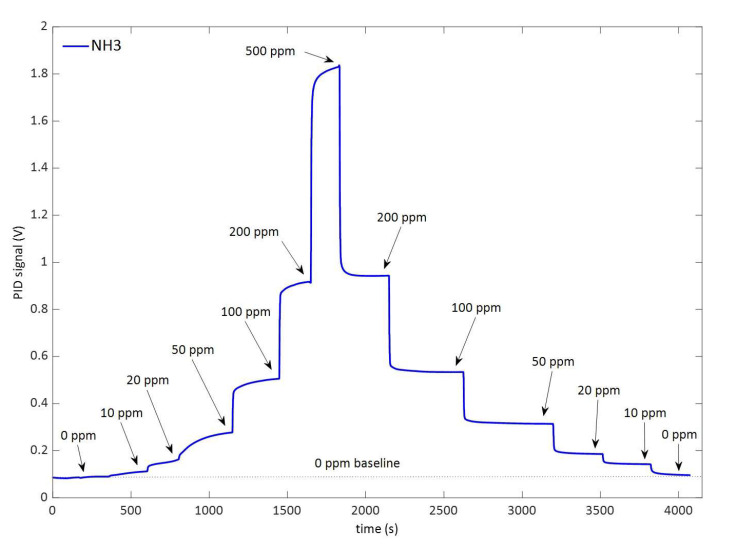
Response of the PID following the variation over time of the concentration of ammonia.

**Figure 9:**
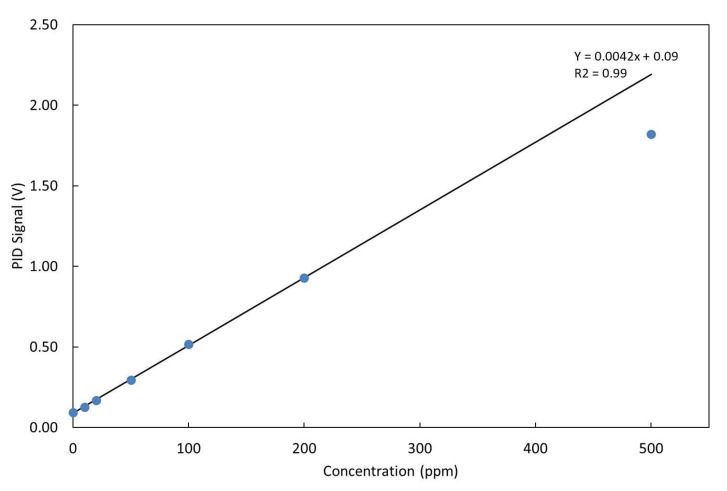
Response of the PID at different concentrations of NH_3_ and linear fit of data up to 200 ppm.

The LIRAS sensor was managed by means of the GUI running on a notebook and was alternately connected to the gas box or to the glass bottle to carry out tests with gases or vapors, respectively. All tests for validation of the LIRAS sensor have been carried out according to the following procedure: the sensor was kept indoors on the test bench and connected to an external power supply unit set at 24 V (HP model E3631A), and to a notebook running the sensor GUI through USB connection, as depicted in Figure 7. In tests with gases, one reference gas cylinder and the nitrogen gas cylinder were connected to the gas box on different inlets. The gas box outlet was directed to the sensor sampling probe, which was a simple Teflon tube, without sealing to avoid overpressure towards the sensor inlet. The mass flow controller and the switching valves of the gas box were set to produce the mix at the desired concentration for the test. Whatever the gas mix, the output flow of the gas box was always set higher (200 mL/min) than the sampling flow of the sensor (100 mL/min), in order to avoid additional dilution of the sampled mix. If considering liquid VOCs, a small amount of liquid (microliters) was injected into the 5.5-L glass bottle depicted in Figure 7. After waiting for a few minutes, to allow almost complete evaporation of the liquid, the bottle was connected by Teflon tubing to the sensor to start sampling. Once the gas/vapor mix was ready for testing, the operator started the sampling and analysis procedure via the GUI of the sensor, and the results were shown in real time on the notebook screen and recorded on file. Figure 8 shows the response over time of the PID in concentration monitoring mode while the sensor was sampling different concentrations of ammonia generated by means of the gas box. Figure 9 shows the same results plotted as a function of the concentration. A detection capability down to 10 ppm and a linear response at least up to 200 ppm were obtained. In this range of linear response, a sensitivity of about 4.2 mV/ppm was evaluated from acquired data. If assuming a conservative 0.5 mV noise level of the PID signal, a LoD of about 100 ppb was estimated. It is useful to point out that the photoionization efficiency of ammonia at 10.6 eV is rather low, thus the ppb detection limit of the mini-PID is not applicable for this molecule, nevertheless this LoD for ammonia is well suited for security applications, because it is well below the safety level of the ERPG-1 [22] limit for ammonia of 25 ppm. The ERPG-1 limit is defined in the Emergency Response Planning Guidelines as the most restrictive limit to avoid adverse health effects from exposure to certain airborne chemical concentrations.

**Figure 10:**
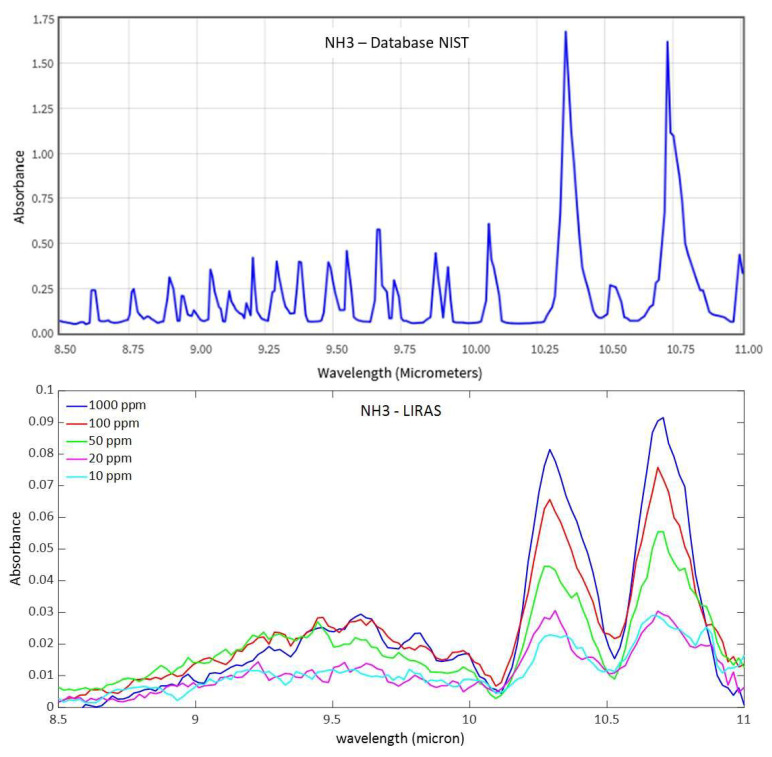
Comparison between the reference spectrum of ammonia from the NIST database (at 4 cm^–1^ resolution) and spectra acquired at different concentrations of ammonia by the LIRAS sensor (at a lower resolution).

Despite the sufficient sensitivity demonstrated to detect ammonia, the PID is intrinsically sensitive to many other molecules (e.g., all the gas molecules that can be ionized by the PID’s lamp, including non-hazardous species) and cannot also be used to identify the presence of a specific hazardous molecule. The miniaturized infrared analyzer of the LIRAS sensor allowed us to overcome this limitation. Within the working spectral range of the analyzer, the LIRAS sensor measures the IR absorption spectrum of the sampled molecule and makes a comparison with a reference database for identification. In Figure 10, a spectrum of ammonia from the NIST reference database of IR spectra [23] and the spectra measured by the LIRAS sensor at different concentrations of ammonia above the detection limit of the PID are shown.

The LIRAS sensor started to resolve the main absorption bands in the spectrum of ammonia at around 10.35 μm and 10.75 μm at the concentration of 10 ppm, which is still below the ERPG-1 level for ammonia. Looking at the plot of measured absorbance in Figure 10, it is worth noting that the response was not linear with the concentration of the sample. This can be explained by the reduced trapping efficiency of the MEMS pre-concentration cartridge when sampling higher concentrations, due to partial saturation of the sorbent material.

**Figure 11:**
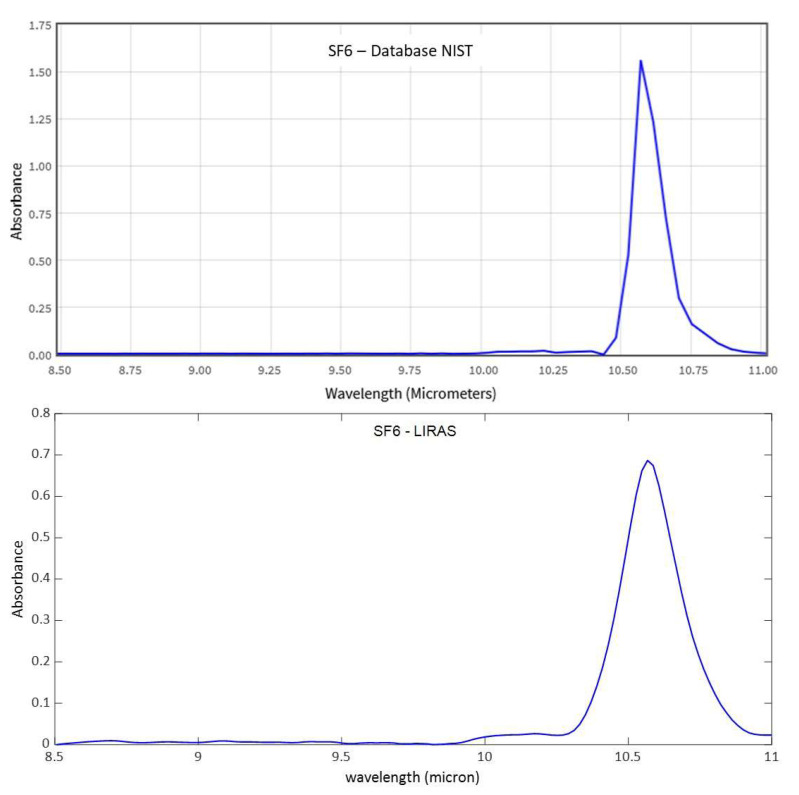
Comparison between a reference spectrum of sulfur hexafluoride from the NIST database (at 4 cm^–1^ resolution) and spectrum acquired by the LIRAS sensor for SF6 at 100 ppm.

**Figure 12:**
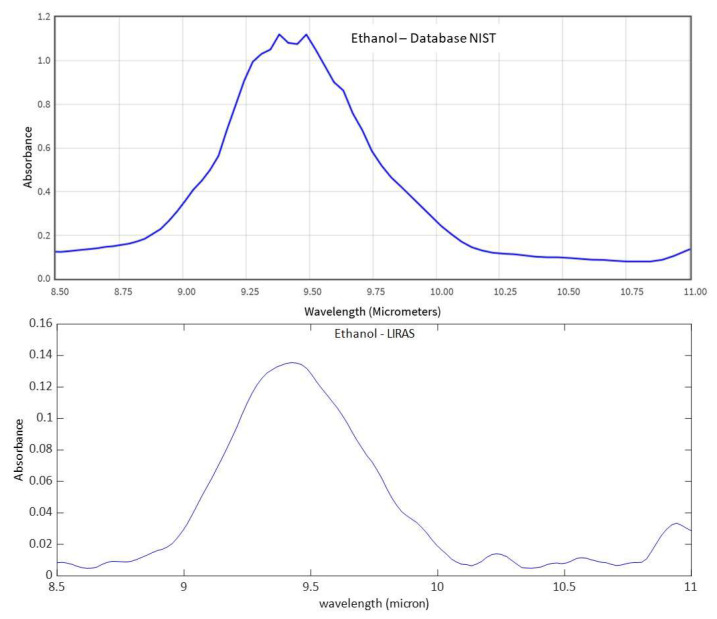
Comparison between the reference IR absorption spectrum of ethanol from the NIST database and the spectrum measured by the LIRAS sensor.

**Figure 13:**
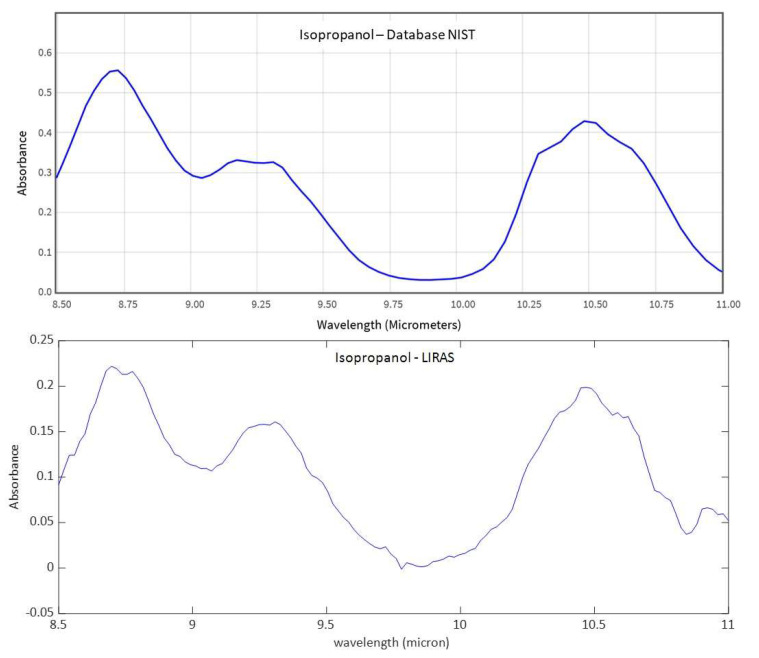
IR comparison between the reference IR absorption spectrum of isopropanol from the NIST database and the spectrum measured by the LIRAS sensor.

Figure 11, Figure 12 and Figure 13 show analogue results for sulfur hexafluoride, ethanol, and isopropanol, respectively. The LIRAS sensor was able to resolve the main absorption bands of all the tested compounds, despite the lower spectral resolution of measured spectra with respect to the reference spectra of the NIST database. However, since the spectral resolution of the LIRAS sensor did not resolve narrow bands, as for the narrower peaks of NH_3_, or broaden them, as for the two peaks of NH_3_ and the main peak of SF_6_, the limit of identification (LoI) of the LIRAS sensor was further investigated. The criterion implemented in the algorithm for automatic identification was to detect the main absorption peaks above the level of noise, and to obtain a Pearson correlation coefficient between measured and reference spectra > 0.8. With this criterion, the sensor was prone to fail the identification, in particular of ammonia. This can be explained by the lower resolution of the LIRAS spectra with respect to the reference spectra in the NIST database. Despite the detection of main peaks, the LIRAS sensor did not resolve narrow peaks, thus often giving correlation coefficients below the selected threshold of 0.8, in particular at low concentrations.

**Figure 14:**
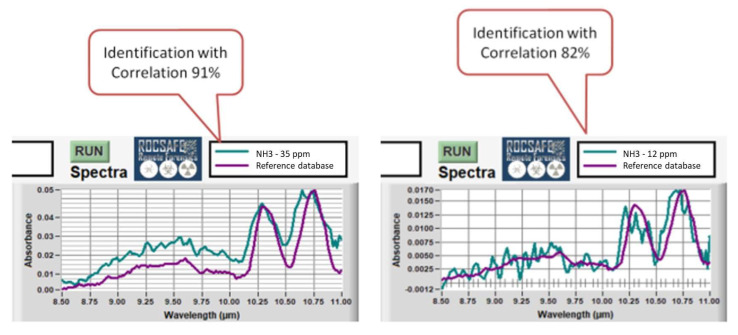
Screenshots of the LIRAS sensor’s GUI showing the identification of ammonia at 35 ppm (on the **left**) and at 12 ppm (on the **right**).

Some LIRAS reference spectra of ammonia were acquired and stored in the sensor experimental database to improve the identification capability. Then, several tests were carried out with ammonia at descending concentrations, and the correlations between measured and reference spectra were computed for each test. Figure 14 shows two screenshots of the LIRAS sensor’s GUI developed for ROCSAFE with the results obtained at the concentrations of 35 ppm and 12 ppm, where the spectrum of ammonia was identified with a correlation of 0.91 and 0.81, respectively. 

An LoI of about 10 ppm was estimated for ammonia. It is worth noting that spectral resolution and working spectral range could be changed by implementing different diffraction gratings and LDAs of different sizes in order to improve the LoI for a targeted molecule.

**Figure 15:**
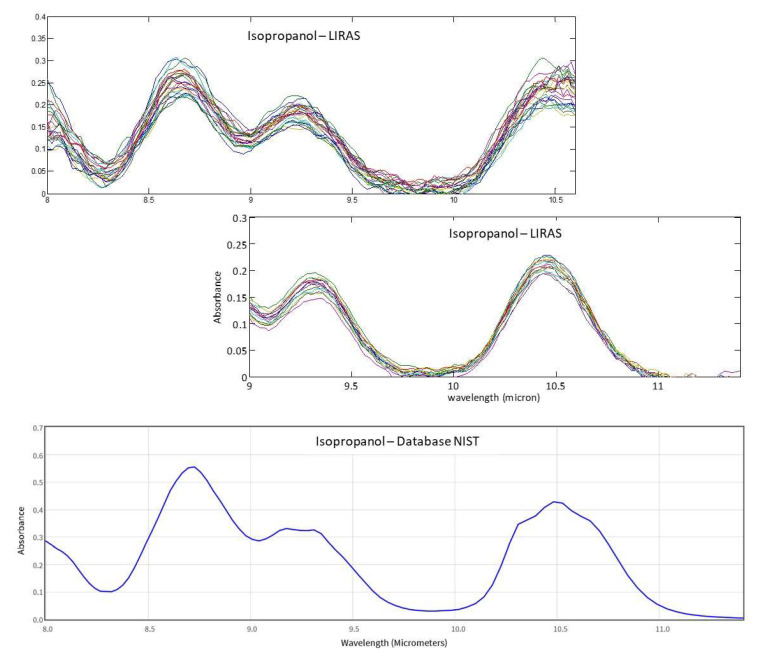
IR absorption spectra of isopropanol measured with the LIRAS sensor tuned at different spectral ranges and the corresponding cumulative reference spectrum from NIST database.

Moreover, the possibility to change the spectral range of the LIRAS sensor was demonstrated by slightly rotating the grating, so as to capture different spectral intervals on the LDA. This allows adaptation of the working spectral range of the LIRAS to acquire the absorption bands of different target molecules of interest in different applications. Compatibly with the transmittance range allowed by the window of the LDA, an extended working spectral range from 8 μm to 11.4 μm was validated, by comparing the measured spectra with the reference spectra of the NIST database. Figure 15 shows, as an example, several spectra of isopropanol acquired in the spectral ranges 8–10.6 μm and 9–11.4 μm. They are compared with the NIST reference spectrum of isopropanol to demonstrate the correspondence in the whole spectral range of 8–11.4 μm.

The LIRAS sensor was also benchmarked with other COTS detectors for chemical threats, namely the model ChemPro 100i [24] by Environics, and the MultiRAE Plus [25] by RAE Systems equipped with a H_2_S sensor and PID, which are reference instruments utilized by first responders for detection of CWAs and TICs. The results of the benchmarking carried out within ROCSAFE are summarized in Table 8. The results show that the LIRAS: (1) was as sensitive as the two conventional chemical detectors; (2) was more selective, since it correctly identified the exact chemical (while tested COTS detectors identified classes, e.g., TIC); (3) gave no false positives; (4) gave specific chemical identification provided that the target substance yielded an IR spectrum in its working spectral range.

## 4. Conclusions

A compact and lightweight LIRAS sensor for gas analysis was developed and validated within the EU project ROCSAFE. The sensor complies with severe constraints on size, weight, and power consumption to be operated onboard UAVs by remote control. The LIRAS sensor was intended to be used for the determination of ‘hot spots’, allowing a more rapid assessment of the scene in the aftermath of an accident and effective collection of forensic evidence. In particular, the LIRAS sensor was dedicated to the detection and identification of TICs and VOCs present in the area by means of air sampling and spectroscopic analysis of the sample. An LoD below the ppm level and identification capability towards several VOCs and TICs including ammonia, sulfur hexafluoride, ethanol and isopropanol were successfully achieved. An LoI of about 10 ppm was achieved for ammonia, which is well suited for prompt identification of this harmful gas even below the restrictive safety level fixed by the ERPG-1 limit of 25 ppm. The benchmarking carried out with reference instrumentation for first responders gave comparable results in terms of sensitivity and even a better performance for the LIRAS sensor in terms of identification capability of NH_3_ and SF_6_, because it always identified the exact compound, while the other benchmarking instrumentation gave only class identification. An improvement of the spectral resolution and an extension of the working spectral range of the LIRAS sensor are foreseen by implementing different gratings and a larger LDA.

## Figures and Tables

**Table 1:** Specifications of the lightweight C sensor.

Housing	Plastic, IP43 (optional)
Size	(25 × 13.5 × 10) cm^3^ (including housing); (20 × 10 × 9) cm^3^ (naked)
Weight	1 kg (including housing), 0.5 kg naked
Power supply	24 Vdc, 15 W peak, <5 W typical
N. of spectral channels	128
Working spectral range	About 2.5 μm interval within 8.0–11.4 μm
Sensitivity	1–10 s ppm (depends on the analyte)
LOI	10 ppm for ammonia
Chemical range	Vapor compounds with fingerprints in the IR working spectral range
Specificity	Discriminates VOCs with spectra in a DB
Operation modes	Concentration monitoring; analysis
Response time	3 s for concentration monitoring with PID; 2–3 min for sampling and IR analysis

**Table 2:** Specifications of the PID.

Model	mini-PID 2
Response time	3 s
Detection limit	In the low ppb range
Response linearity	within 98% over 4 orders of magnitude
Power supply voltage	3.3 V
Power consumption	0.11 W
Weight	<8 g

**Table 3:** Specifications of the 3-way valve.

Model	S070C-SAG-32
Type	Solenoid
N. of ports	3
Power supply voltage	12 V
Power consumption	0.35 W
Weight	5 g

**Table 4:** Specifications of the IR thermal source.

Model	EMIRS-200
Dimension	2.1 × 1.8 mm^2^
Socket	TO39
Buildup	Reflector 1
Parabola power into <20° angle	60%
Window	None
Working Temperature	450 °C at 450 mW
Modulation depth	80% at 10 Hz, 50% at 50 Hz
Emissivity	0.95
Voltage	5.2 V at 450 mW
Current	86 mA at 450 mW

**Table 5:** Specifications of the hollow fiber.

Wavelength range	2.9 μm past 10.6 μm
Length	10 cm
Hollow core diameter	0.5 mm
Material	silica
External coating	Acrylate buffer
Internal coating	Ag/AgI
No end reflection	
Transmission optimized for CO_2_	

**Table 6:** Specifications of the mini-spectrometer’s grating.

Model	GR1325-07106
Type	Ruled reflective
Blaze Angle	21°
Optimum Eff. Range	(9.0–11.0) μm
Grooves/mm	75
Dispersion (nm/mrad)	12.3
Dimension	(25 × 12.5 × 9.5) mm^3^
Clear Aperture	90% of length & width
Surface Quality	60–40 Scratch-Dig
Design Wavelength	10.6 μm

**Table 7:** Specifications of the spectrometer’s linear detector array.

Model	Pyrosens 128-LTI SP0.5 F8-14
Responsivity	5.40 × 10^5^ V/W
Signal	646 mV
Noise	1.48 mV
Size	(0.5 × 0.09) mm^2^
Pitch	0.1 mm
Total length	12.8 mm

**Table 8:** Benchmarking of the LIRAS sensor with COTS detectors.

Agent	Conc.	ChemPro 100i		MultiRAE Plus		LIRAS
	[ppm]	Library: TIC Class.*	Library: FR **	H_2_S Sensor	PID Sensor	
NH_3_	20	No Alarm	No Alarm	NA	NA	Identified
NH_3_	50	Detected	Detected	NA	NA	Identified
NH_3_	100	TIC Hydrite	No Alarm	0	10	Identified
SF_6_	20	Detected	Detected	NA	NA	Identified
SF_6_	50	No Alarm	No Alarm	NA	NA	Identified
SF_6_	100	Detected	Detected	0	0	Identified
PH_3_	5	Detected	Detected	6	1,6	Detected
H_2_S	25	Detected	Detected	8	32	Detected

* Reference library for TIC auto-classifier. ** Reference library for first responders.

## Data Availability

The data presented in this study are available on request from the corresponding author.
